# Resection margins, lymph node harvest and 3 year survival in open and laparoscopic colorectal cancer surgery; a prospective cohort study

**DOI:** 10.1007/s12672-023-00824-x

**Published:** 2023-12-03

**Authors:** M. I. M. De Zoysa, M. D. S. Lokuhetty, S. L. Seneviratne

**Affiliations:** 1 University of Colombo, Colombo, Sri Lanka; 2 Nawaloka Hospital Research and Education Foundation, Colombo, Sri Lanka

## Abstract

**Introduction:**

Laparoscopic colorectal cancer surgery has been gaining popularity in the last decade. However, there are concerns about adequate lymph node dissection and safe resection margins in laparoscopic colorectal cancer surgery. This study was aimed at comparing the lymph node (LN) clearance and surgical resection margins and 3-year survival for open and laparoscopic colorectal cancer surgery.

**Method:**

A pre-tested interviewer administered questionnaire was used to assess the adoption of the laparoscopic approach by Sri Lankan surgeons. Data was collected prospectively from patients who underwent open or laparoscopic colorectal cancer surgery at the University Surgical Unit of the National Hospital of Sri Lanka from April 2016 to May 2019. The histopathology records were analysed to determine the longitudinal and circumferential resection margins(CRM) and the number of lymph nodes harvested. The resection margins were classified as positive or negative. The total number of LN examined was evaluated. Presence of local recurrence and liver metastasis was determined by contrast enhanced CT scan during 3-years of follow up. Chi square, T test and z test for proportions were used to compare CRM, LN harvest and survival rates between the groups.

**Results:**

Of the surgeons interviewed only 11 (18.4%) performed laparoscopic colorectal cancer surgery. A total of 137 patients (83 males and 54 females) were studied. Eighty-one procedures were laparoscopic and 56 procedures were open. All patients had clear longitudinal resection margins. Seventy-eight patients in the laparoscopic group (96%) and 51 patients (91%) in the open group had clear CRM (p > 0.05). A total of 2188 LNs (mean 15.9) were resected in all procedures. Six-hundred-eighty-nine lymph nodes were removed during open procedures (mean 12.3, SD 0.4) and 1499 (mean 18.5, SD 0.6) were removed during laparoscopy (p < 0.05). At 3 years follow-up the disease-free survival in the laparoscopic and open colon cancer patients was 27/41 (65.8%) and 16/29 (55.1%) respectively (p = 0.35). Disease free survival in the laparoscopic and open rectal cancer patients was 23/38 (60.5%) and 13/25 (52.0%) respectively (p = 0.40). Four patients were lost during follow-up.

**Discussion and conclusion:**

CRM was comparable in the two groups. Laparoscopic group had a significantly higher LN harvest. Three-year survival rates were similar in the two groups. Acceptable results can be obtained with laparoscopic colorectal cancer surgery.

## Introduction

Most abdominal surgical procedures can be performed laparoscopically. Laparoscopic surgery has the advantages of less post-operative pain, early ambulation, shorter hospital stay, early return to normal activity and smaller surgical scars. In most countries including South Asian countries like Sri Lanka over 95% of cholecystectomies are performed laparoscopically. In western countries over 60% of colorectal cancer surgeries are performed laparoscopically [1–3]. In countries like Sri Lanka the rates of laparoscopic surgery for CRC are much lower probably due to concerns about tumour and nodal clearance of the laparoscopic approach and patient survival.

We did a survey among surgeons who perform colorectal surgery to determine the adoption of the laparoscopic approach. We also analyzed two cohorts of consecutive patients undergoing open and laparoscopic colorectal cancer surgery to compare the lymph node (LN) clearance and surgical resection margins for open and laparoscopic colorectal cancer surgery and determined their 3-year and disease free survival.

## Methods

1. A telephone survey was carried out among 59 randomly selected surgeons employed in Government hospitals in Sri Lanka who operate on CRC patients. A pre-tested questionnaire was used to gather information on each surgeon’s use of the laparoscopic approach. All those interviewed were specialist consultant surgeons. The same interviewer interviewed all the surgeons using the same structured questionnaire.

Data from consecutive patients who underwent open and laparoscopic colorectal cancer surgery at the university surgical unit of national hospital of Sri Lanka from April 2016 to May 2019 was analyzed. Patients were enrolled prior to surgery and data was collected by a research assistant. The details of surgery were obtained from the patient’s operation notes. The details about longitudinal and circumferential resection margins and the number of lymph nodes harvested were obtained from the histopathology reports. All data was entered into a SPSS database.

The resection margins were classified as positive or negative. The total number of LN examined was evaluated. Chi square test was used to compare CRM and student T test was used to compare the LN harvest. All enrolled patients were operated and by the same surgeons and histopathological reporting was done by the same group of pathologists. Patients underwent CEA estimations and ultrasonography every six months and colonoscopies annually. Contrast enhanced CT scans of the chest and abdomen were performed in patients who had liver lesions suspicious of metastasis of metastasis, elevated CEA levels or evidence of anastomotic recurrence on colonoscopy. The z test was used to compare the overall survival and disease free survival rates between the groups of patients.

## Results

Of the 59 surgeons who took part in the survey, there were 48 (81.3%) general surgeons, 6 (10.1%) GI surgeons and 5 (8.6%) oncological surgeons. Facilities for laparoscopic surgery were available to 53 (89.8%) surgeons. Forty general surgeons (95.2%) and all GI surgeons performed laparoscopic cholecystectomy. Facilities for colorectal cancer surgery (including a suitable energy source) were available to 46 (77.9%) surgeons which included all GI and oncological surgeons and 35 general surgeons. Of the general surgeons only 4 (11.4%) performed laparoscopic colorectal cancer surgery. Four (67%) GI surgeons and 3 (60%) oncological surgeons performed laparoscopic CRC surgery. The reasons given for performing laparoscopic surgery were: non-availability of a laparoscope and/or a suitable energy source longer operating time, heavy workload, inadequate training and non-availability of trained assistants.

There was a total of 137 colorectal cancer patients [83 males (60.5%) and 54 females (39.5%)] who underwent surgical resection. The mean age was 58.6 years. (SD ± 10.2) (Male age range 34–80 years; female age range 40–78 years). Out of 137 patients 81 patients (59.1%) underwent a laparoscopic resection (36 female and 45 male patients) and 56 (40.9%) consecutive patients had an open procedure (38 male and 18 female patients). Four patients with colon cancer and 5 patients with rectal cancer were converted from laparoscopic to open surgery. As the converted patients had both extensive laparoscopic and open dissection, they could not be included in either category and they were excluded from the study.

Table 1 illustrates the type of operation performed.

**Table 1:** Patient characteristics and surgical procedures

	Laparoscopic group	(%)	Open group	(%)
**Sex**				
Male	45	32.8	38	27.7
Female	36	26.2	18	13.2
**Surgical procedures**				
Right hemicolectomy	10	7.2	07	5.1
Left hemicolectomy	15	11.0	08	5.8
Sigmoid colectomy	17	12.4	15	11.0
Anterior resection	37	27.0	22	16.0
Abdominal perineal resection	02	1.5	04	2.9

All patients had clear longitudinal resection margins. 78 patients in the laparoscopic group (96%) and 51 patients (91%) in the open group had clear CRM. There was no significant difference between open and laparoscopic group (p > 0.05).

A total of 2188 LNs (mean 15.9) were resected in all procedures. In open procedures 689 LNs were removed (mean 12.3, SD 0.4) and 1499 LNs were removed (mean 18.5, SD 0.6) during laparoscopic procedures. The mean number of LN resected in laparoscopic procedures was higher and differences between the open and laparoscopic groups were statistically significant (p < 0.05).

The total number of LN harvested from laparoscopic and open colon cancer patients was 818 (mean − 19.47) and 396 (mean − 13.2) respectively and the difference was statistically significant (p < 0.05).

The total number of LN harvested from laparoscopic and open rectal cancer patients was 681 (mean − 17.46) and 293 (mean − 11.2) respectively and the difference was statistically significant (p < 0.05).

At 3 years follow-up the overall survival in the laparoscopic and open colon cancer patients was 36/42 (85.7%) and 22/30 (73.3%) respectively (p = 0.19). Overall survival in the laparoscopic and open rectal cancer patients was 30/39 (76.9%) and 16/26 (61.5%) respectively (p = 0.18).

At 3 years follow-up the disease-free survival in the laparoscopic and open colon cancer patients was 27/41 (65.8%) and 16/29 (55.1%) respectively (p = 0.35). Disease free survival in the laparoscopic and open rectal cancer patients was 23/38 (60.5%) and 13/25 (52.0%) respectively (p = 0.40).

## Discussion

Our survey among practicing surgeons in Sri Lanka showed that less than one fourth of the surgeons who had the necessary facilities performed laparoscopic surgery for CRC. As these laparoscopic surgeons also performed open procedures the actual laparoscopic colectomy rates would be much lower than the percentage of surgeons who perform laparoscopic procedures for CRC. In South Asian countries like Sri Lanka the laparoscopic colectomy rates remain low due to many reasons. A large study from a high-volume center in India [4] and a smaller study from Sri Lanka [5] have shown that nearly 80% of tumors are advanced at the time of presentation. One of the reasons for low laparoscopic colectomy rates in this part of the world is concern about the adequacy of locoregional clearance in these advanced tumors. Other reasons include lack of adequate training and facilities for advanced laparoscopic procedures. In contrast there is a steady increase in the adoption of laparoscopy for CRC surgery worldwide with the US showing an increase from 22.7% to 49.8% from 2007 to 2014 [1]. In the UK there has been an increase in laparoscopic CRC resections 48% to 61% from 2013/14 to 2019 [2]. In Netherlands over 60% of patients undergo laparoscopic resections for CRC [3]. In our study we compared our first 81 patients who underwent laparoscopic surgery with the last 56 patients who underwent open surgery. Although the surgeons who performed these procedures were much more experienced in open surgery the laparoscopic surgery group had a significantly higher LN harvest than the open group. This difference may be due to better visualization and skeletonization of the pedicles in laparoscopic group. The retrieved and number of assessed lymph node harvest in majority of patients in both groups was higher than the threshold of 12 lymph nodes, recommended by the American Joint Committee on Cancer (AJCC) [6]. Furthermore, in our study the longitudinal margins and CRM were comparable in the two groups. Overall, our findings emphasized that adequate tumour margin and better lymph node harvest can be obtained with laparoscopic colorectal cancer surgery in both colonic and rectal cancer surgery.

Our study was not a randomized controlled trial. However, the histo-pathological findings would not have been significantly affected, as the pathologists who examined the specimens were not aware whether the operation was open or laparoscopic. A few previous studies have shown that the mean number of harvested lymph nodes was significantly higher in laparoscopic than in open surgery [7–9]. Our findings are similar to theirs. However, some studies have shown no difference between the number of LN resected in open and laparoscopic surgery [10–12]. It is not clear whether the two groups of patients included in these previous studies were operated on by the same surgeons.

In our study the longitudinal margins and CRM were comparable in the laparoscopic and open surgery groups. A few previous studies have also shown comparable CRM in LR and OR, with no positive distal margins in either group [9, 13]. Our findings are very similar to theirs.

Most previous studies have compared either the lymph node harvest or the resection margins in LR and OR. Both these aspects were evaluated in our study. Both CRM and LN clearance are important for tumour staging and prognostication at the time of surgery. In our study a single surgical team operated on all patients and processing and examination of resected specimens was done by a single histopathology team thus eliminating undue bias. Patients were followed up for 3 years and there was no significant difference in both overall and disease free survival between the two groups. Previous studies have shown comparable recurrence and survival rates in laparoscopic and open surgery [12–14]. The significantly better LN clearance in our study could be attributed to the more precise high resolution and brighter image which allows the surgeon to perform a more radical and precise resection of the mesocolon and mesorectum, while facilitating an accurate and complete lymphadenectomy with higher transection of the vascular pedicles. Earlier studies may have failed to show better LN clearance in LR due to poorer optics at the time these studies were done.

In spite of the knowing the benefits of laparoscopic surgery for CRC the majority of surgeons practicing in Sri Lanka showed a reluctance to adopt this approach. One of the reasons was their concern about the adequacy of adequate locoregional tumour clearance and survival in view of the fact that tumours in this part of the world are more advanced at the time of presentation. Our study showed that comparable tumour clearance, better nodal clearance and acceptable survival rates could be obtained in laparoscopic colorectal cancer surgery. The findings of this study are likely to encourage more South Asian surgeons to adopt the laparoscopic approach thereby increasing the proportion of laparoscopic procedures performed which would result in better patient outcomes.

In conclusion, this study highlights the fact that satisfactory oncological outcomes including clear margins, lymph node harvest and survival rates can be obtained with laparoscopic colorectal cancer surgery in a South Asian developing country.

## Data Availability

All data and materials are available with the corresponding author.
